# New layers of regulation of the general stress response sigma factor RpoS

**DOI:** 10.3389/fmicb.2024.1363955

**Published:** 2024-03-05

**Authors:** Simon Handler, Clare L. Kirkpatrick

**Affiliations:** Department of Biochemistry and Molecular Biology, University of Southern Denmark, Odense, Denmark

**Keywords:** sigma factor, GSR, phenotypic heterogeneity, *E. coli*, RpoS, gene regulation

## Abstract

The general stress response (GSR) sigma factor RpoS from *Escherichia coli* has emerged as one of the key paradigms for study of how numerous signal inputs are accepted at multiple levels into a single pathway for regulation of gene expression output. While many studies have elucidated the key pathways controlling the production and activity of this sigma factor, recent discoveries have uncovered still more regulatory mechanisms which feed into the network. Moreover, while the regulon of this sigma factor comprises a large proportion of the *E. coli* genome, the downstream expression levels of all the RpoS target genes are not identically affected by RpoS upregulation but respond heterogeneously, both within and between cells. This minireview highlights the most recent developments in our understanding of RpoS regulation and expression, in particular those which influence the regulatory network at different levels from previously well-studied pathways.

## Introduction

Sigma factors are proteins that bind to the RNA polymerase (RNAP) core enzyme to make the holoenzyme, which directs transcription of a specific set of genes by allowing RNAP to initiate transcription of different classes of promoters. Bacteria encode so-called “housekeeping” sigma factors that allow the expression of genes necessary for maintaining normal cellular functions, but also “alternative sigma factors” that control the transcription of fewer, but more specific genes. In *E. coli*, one housekeeping sigma factor called σ^70^ or σ^D^, as well as six alternative sigma factors called σ^E^, σ^F^, σ^H^, σ^I^, σ^N^, and σ^S^ have been identified. These alternative sigma factors play an essential role in the induction of genes during bacterial stress, such as iron or nitrogen depletion (σ^I^ and σ^N^) or heat shock (σ^E^ or σ^H^). In *E. coli* however, the most important alternative sigma factor for general stress tolerance is σ^S^, also termed RpoS or σ^38^ (annotated as RpoS throughout this review). This sigma factor is encoded by the *rpoS* gene and allows *E. coli* to simultaneously respond to a large variety of stresses, a response termed the “general stress response” (GSR). Induction of the GSR not only allows the cells to become resistant to a specific stress but also mediates cross-resistance to other stresses. For example, cells that are starved for carbon also become resistant to hydrogen peroxide, high temperatures and low pH. The ability of the cells to respond to such a large repertoire of stresses requires transcription of multiple genes, and in fact, the RpoS regulon constitutes about 500 genes, which corresponds to 10% of the *E. coli* genome. Thus, RpoS is regarded as being the master regulator of the GSR in *E. coli* (Battesti et al., 2011; Landini et al., 2014; Gottesman, 2019).

**Figure 1:**
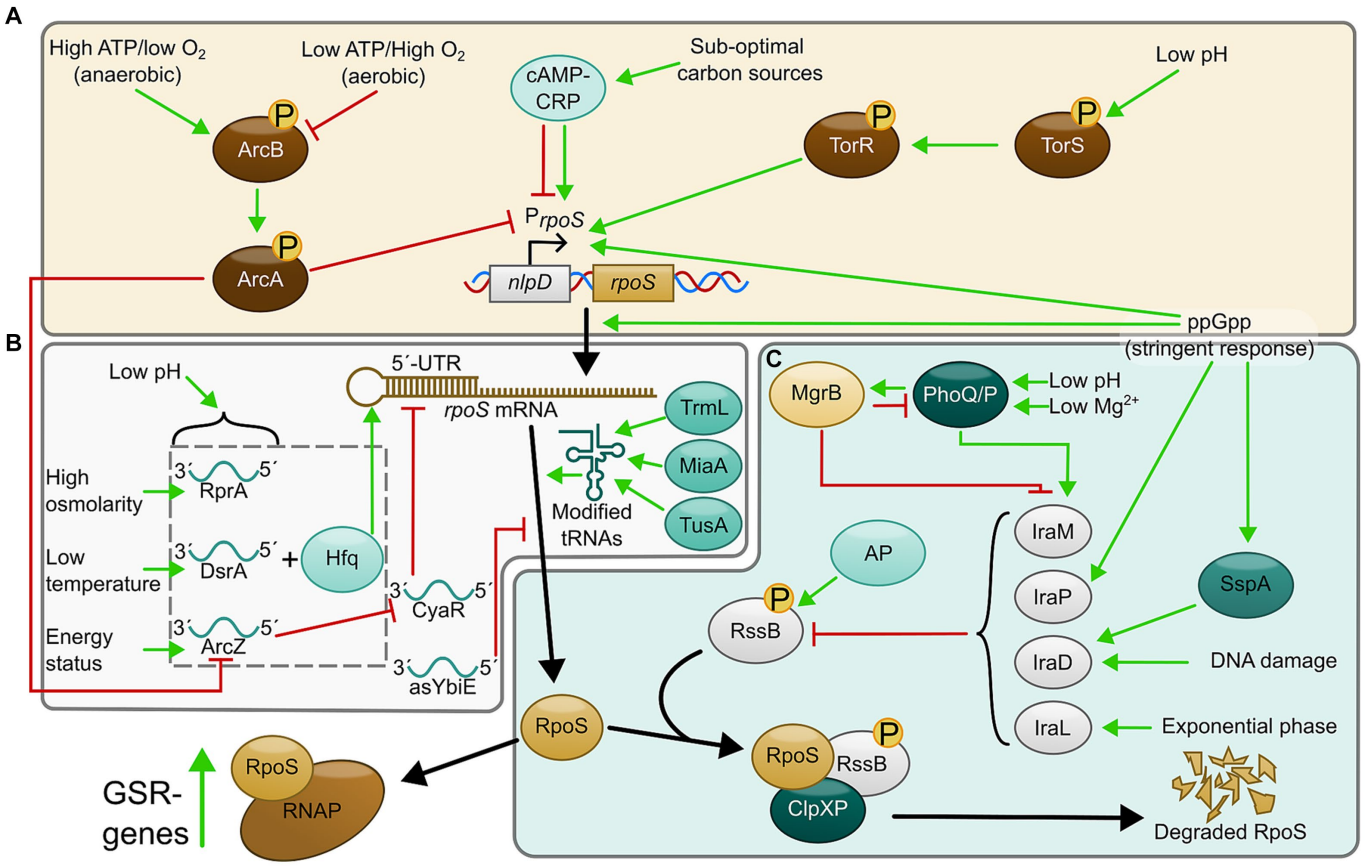
Regulatory pathways controlling production and activity of RpoS. **(A)** Transcriptional regulation, **(B)** translational regulation, and **(C)** regulation of stability and degradation. AP, acetyl phosphate.

Under optimal laboratory conditions or in exponential phase, the levels of RpoS are generally very low, but increase as the cells enter stationary phase. During stationary phase, growth rate becomes slower due to limitations of nutrients such as carbon, nitrogen, and ATP, as well as due to the generation of excessive amounts of reactive oxygen species (ROS). Thus, RpoS levels rise in these conditions to express the necessary enzymes and defense systems before the nutrients and building blocks of metabolism become too low for the cells to survive. Such a wide response to multiple stresses requires multifactorial regulation of RpoS. Indeed, RpoS is regulated at the transcriptional, translational, and post-translational level (Figure 1). Recently, additional regulatory factors have been discovered that modulate the downstream effects of RpoS depending on target promoter sensitivity to RpoS levels (Wong et al., 2017), proportion of rare codons in the RpoS-target genes (Aubee et al., 2016, 2017), and ribosome heterogeneity (Kurylo et al., 2018). This review will cover how RpoS production and activity is regulated in *E. coli* and the result of these pathways on the bacterial GSR, including the possible implications of heterogeneity-inducing mechanisms.

## Multi-level regulation of RpoS allows for co-ordination of multiple signal inputs

### Regulation of transcription

*rpoS* transcription is under control of two major promoters: one upstream of the *nlpD* gene and one intragenic promoter within *nlpD*. The upstream promoter gives rise to a *nlpD-rpoS* bistronic transcript transcribed at low levels during exponential growth (Lange and Hengge-Aronis, 1994b; Landini et al., 2014). The intragenic promoter (hereafter referred to as P*_rpoS_*) is considered the most important as it regulates *rpoS* transcription during stress (Lange et al., 1995; Landini et al., 2014). In addition to these, four alternative transcription start sites have been located very close to the start codon of the *rpoS* gene and another upstream of *nlpD* (Mendoza-Vargas et al., 2009), although it is unknown under what conditions these are used. P*_rpoS_* is regulated by various transcription factors, such as ArcA (Figure 1A). ArcA is a cytoplasmic response regulator of a two-component system called ArcAB, where ArcB is the membrane-anchored histidine sensor kinase. ArcAB regulates P*_rpoS_* according to the aerobic status of the environment, such that it is expressed under anaerobic conditions (Battesti et al., 2011). Intriguingly, Henge et al. found that P_rpoS_ contained a binding site for phosphorylated Arc-P, making it so that during anaerobic conditions, reduced quinones activate autophosphorylation of ArcB to phosphorylate and activate ArcA. ArcA-P will then repress P_rpoS_ by direct binding. In contrast, during aerobic conditions, oxidized quinones interfere with the ArcB autophosphorylation (Alvarez et al., 2013), resulting in a lower amount of ArcA-P and a de-repression of P_rpoS_ (Georgellis et al., 2001; Malpica et al., 2004; Mika and Hengge, 2005). A binding site for the global regulator CRP (a regulator that controls gene expression in response to sub-optimal carbon sources (Gosset et al., 2004; Landini et al., 2014), was also identified in P_rpoS_ although whether CRP is a positive or negative regulator for this promoter is still under debate (and likely growth phase-related; see Gottesman (2019)) for review of the current state of knowledge). P*_rpoS_* is also regulated by nutritional status of the cell signaled by ppGpp (Lange et al., 1995; Brown et al., 2002). ppGpp production mediates downregulation of rRNA biosynthesis, ribosome production and tRNA production, as well as inducing genes required for amino acid biosynthesis and uptake (Magnusson et al., 2005; Srivatsan and Wang, 2008). In *E. coli*, the increase of RpoS as cells enter stationary phase correlates with an increase of ppGpp (Gentry et al., 1993), as a result of carbon, nitrogen, phosphate, fatty acid and/or iron starvation (Spira et al., 1995; Brown et al., 2002; Vinella et al., 2005; Srivatsan and Wang, 2008; Traxler et al., 2008).

A recent paper has shown that the TorR/TorS two-component system is also involved in the transcriptional regulation of *rpoS* in response to extremely acidic environments. TorS is the histidine kinase that phosphorylates TorR (response regulator) during this response. Interestingly, both TorR (Li and Yao, 2022) and ArcA (Mika and Hengge, 2005) could be phosphorylated in the absence of their cognate sensor kinases, suggesting that signal inputs from multiple sources could be integrated via these two response regulators.

### The importance and versatility of small RNAs’ (sRNAs) control of RpoS levels

The transcription start site of *rpoS*, when transcribed from P_rpoS_, is located 567 nt upstream of its start codon, giving it a long 5′ untranslated region (5’-UTR) (Landini et al., 2014). When *rpoS* is transcribed, this 5’-UTR folds into a stem-loop that inhibits ribosome binding, preventing the translation of *rpoS* mRNA (Brown and Elliott, 1997; Cunning et al., 1998; Battesti et al., 2011). The inhibitory structure of this stem-loop is overcome by sRNAs binding to a “hub” on the 5’-UTR, resulting in the opening of the stem-loop allowing ribosome binding and translation (Figure 1B). Three sRNAs involved in this regulation are ArcZ, DsrA, and RprA which respond to energy status, low temperature and osmolarity stress, respectively, (Sledjeski et al., 1996; Muffler et al., 1996c; Majdalani et al., 1998, 2001, 2002; Mandin and Gottesman, 2010; Battesti et al., 2011). These sRNAs require a chaperone protein called Hfq for their action, which stabilizes the sRNAs and promotes pairing with mRNA targets (Muffler et al., 1996b; Landini et al., 2014). However, any transcripts produced from the alternative transcription start sites mentioned previously would completely lack the 5’-UTR and therefore be insensitive to sRNA-mediated regulation, potentially allowing the cell to bypass this mechanism under some conditions.

ArcZ provides an auto-regulatory loop functioning as a homeostatic regulator for the ArcA/ArcB two-component system. Phosphorylated ArcA has been shown to repress *arcZ* expression when ATP levels are high, and dephosphorylated ArcA mediating de-repression when energy levels are low (Mandin and Gottesman, 2010). The same study also showed that the ArcZ and ArcB transcripts negatively regulate each other, suggesting that the role of ArcZ is to provide negative feedback to ArcAB by decreasing the levels of ArcA-P, effectively maintaining the expression of *rpoS* mRNA during aerobic conditions, while during anaerobic conditions, ArcB transcripts would inhibit ArcZ, to maintain effective inhibition of P_rpoS_ by ArcA-P. Thus, ArcZ is an example of a functional link of both the transcriptional and translational regulation of RpoS (Mandin and Gottesman, 2010). Furthermore, ArcZ also seems to be involved in the response to dehydration stress, suggesting that ArcZ could be one of the factors promoting induction of RpoS to enhance expression of genes essential for survival in environments with low water activity (Chen and Goulian, 2018).

DsrA was originally found to be important for *E. coli*’s response to cold stress by upregulating RpoS during the exponential phase (Sledjeski et al., 1996; Repoila and Gottesman, 2001), but a later paper also characterized DsrA, RprA and to some extent ArcZ to be involved in the acid stress response in *E. coli* (Bak et al., 2014). Intriguingly, Kim et al. found that DsrA to some extent could increase *rpoS* levels without the need of Hfq (Kim et al., 2019). Although the exact mechanism is unknown, stabilization of *rpoS* mRNA by direct binding of DsrA, as well as suppression of Rho-dependent transcription termination were some of the suggested explanations (Sedlyarova et al., 2016; Kim et al., 2019). A recent study found that DsrA in complex with Hfq transiently samples the nascent *rpoS* mRNA during the transcription. Hfq-DsrA was shown to bind 4 times faster to *rpoS* during its transcription compared to refolded *rpoS*, suggesting that sRNAs that target the *rpoS* transcript as it emerges from RNAP provides a kinetic advantage to the following translational regulation (Rodgers et al., 2023).

A recent study elucidated the role of CyaR, an sRNA that unlike ArcZ, DsrA and RprA is a negative regulator of *rpoS* translation (Kim and Lee, 2020). This sRNA was shown to interact with ArcZ, in addition to *rpoS*, so that the ArcZ-CyaR complex relieves CyaR inhibition of *rpoS* (Kim and Lee, 2020). Another study developed a method termed “rGRIL-seq” that allowed the identification of direct targets of sRNAs in living cells. Using this method, another regulatory sRNA called “asYbiE” was identified. This sRNA is encoded (antisense) within the ORF of the *ybiE* gene, and likely represses *rpoS* translation by base-pairing with the Shine-Dalgarno sequence of *rpoS* (Han and Lory, 2021). Other sRNAs that regulates *rpoS*-translation include OxyS and MgrR (Landini et al., 2014; Han and Lory, 2021). OxyS is induced by oxidative stress, such as increased hydrogen peroxide levels, and modulates RpoS levels by binding to Hfq, preventing the interaction of other sRNAs with this protein and repressing *rpoS* translation (Zhang et al., 1998; Mandin and Gottesman, 2010; Moon and Gottesman, 2011). MgrR is an sRNA that was identified to bind to Hfq, and its expression was later shown to be dependent upon PhoQ/PhoP, a TCS that responds to low Mg^2+^ (Soncini et al., 1996; Zhang et al., 2003; Moon and Gottesman, 2009). Although the sRNA has been shown to complex with *rpoS* mRNA and inhibit translation by associating with Hfq and interacting with the 5’-UTR, the physiological role of this interaction is currently unclear (Moon and Gottesman, 2011; Han and Lory, 2021). In conclusion, sRNAs and antisense RNAs are also implicated in negative as well as positive regulation of *rpoS* translation and can modify *rpoS* translation by mechanisms other than 5’-UTR binding/unfolding.

### Anti-adaptor-mediated regulation of RpoS-stability

The RpoS protein is normally rapidly degraded during exponential growth in *E. coli*, but this degradation is relieved during stationary phase or nutrient scarcity (Lange and Hengge-Aronis, 1994a; Mandel and Silhavy, 2005; Battesti et al., 2011). RpoS degradation is mediated through the ClpXP-protease, and the ability of the protease to efficiently degrade RpoS is triggered by the RssB protein, a response regulator that directly binds RpoS to deliver it to the protease (Muffler et al., 1996a; Klauck et al., 2001). RssB levels are controlled by “anti-adaptors” called IraP, IraM and IraD that can modulate the activity of RssB by means of sequestration (Figure 1C). This prevents ClpXP-mediated degradation of RpoS and allows the cell to increase RpoS in response to environmental stress (Bougdour and Gottesman, 2007; Bougdour et al., 2008; Merrikh et al., 2009b).

IraD is induced by DNA-damaging agents like hydrogen peroxide and ROS that accumulate during excess cell growth (Merrikh et al., 2009a), but a recent study found that DnaA is also involved with regulation of IraD (Sass et al., 2022). DnaA-ATP was found to act as a repressor of *iraD* expression by binding to its promoter. Furthermore, this repression was relieved by activation of Hda to induce expression of IraD in response to DNA-damage or a stalled replication (Sass et al., 2022). Interestingly, IraD seems to not only be regulated in response to DNA damage, but also by nutritional stress mediated by ppGpp. *iraD-*levels have been shown to be reduced in ppGpp-compromised mutants, and the response to ppGpp seemed to depend on growth phase (Merrikh et al., 2009b). Sass and colleagues also revealed that SspA, a transcription factor induced by accumulation of ppGpp (Williams et al., 1994), exerts complex regulatory effects on *iraD* expression, with a positive influence on basal transcription but negative when DNA damage is chemically induced (Sass et al., 2022). However, the relationship between SspA and DnaA regulation of *iraD* remains to be determined (Sass et al., 2022).

IraM is an anti-adaptor that induces RpoS stability in response to Mg^2+^ starvation though the upstream PhoP/PhoQ two component system (Minagawa et al., 2003; Zwir et al., 2005; Bougdour et al., 2008). Additionally, IraM also seems to be involved in response to acid stress through MgrB, a membrane peptide functioning as a feedback inhibitor of PhoP/Q (Lippa and Goulian, 2009; Xu et al., 2019). *E. coli* deletion mutants of MgrB showed an increased expression of acid-resistance genes and had increased RpoS levels, but the phenotypes were reversed if *iraM* was deleted as well, suggesting that MgrB negatively regulates expression of RpoS-controlled acid resistance genes by repressing PhoP/Q-mediated induction of IraM (Xu et al., 2019).

IraP mediates stabilization of RpoS by responding to phosphate starvation through ppGpp (Bougdour and Gottesman, 2007). More recently, DksA has been shown to strongly induce RpoS protein levels, in conjunction with ppGpp, by increasing the transcription of the *iraP*-promoter (Girard et al., 2018).

A fourth anti-adaptor called IraL (orthologous to IraM) has also been identified in the *E. coli* CFT073 uropathogenic strain. Similar to the other anti-adaptors, IraL was also identified to bind and sequester RssB, but does so during the logarithmic phase. Thus, an initial stabilization of RpoS during early growth phase could function as a stress-anticipation system in some pathogenic *E. coli* strains (Hryckowian et al., 2014).

Finally, Henge et al. also showed that ArcB is involved in regulating RpoS protein levels, by means of phosphorylation of RssB, activating it to target RpoS for degradation. Thus, the ArcAB TCS also provides a functional link with the transcriptional and post-translational regulation of RpoS, suggesting that *E. coli’*s response to oxygen levels is of utmost importance for its viability (Mika and Hengge, 2005).

### New regulatory mechanisms contribute to increased network complexity and to downstream heterogeneity of gene expression

In addition to the regulation of RpoS at transcriptional, post-transcriptional and stability levels, tRNA-dependent RpoS translation rate regulation has also been observed. MiaA, a tRNA isopentenyltransferase protein that catalyzes the addition of 2-methylthio-N^6^-(Δ^2^-isopentenyl) or ms^2^i^6^A onto adenine 37 that recognizes codons beginning with uridine (Connolly and Winkler, 1989), was shown to be necessary for the full expression of RpoS by increasing the translation of the *rpoS* reading frame (Thompson and Gottesman, 2014). A further study revealed that the ORFs for both *rpoS* and *iraP* contained an unusually high degree of UUX-leucine codon usage, making them more sensitive to the MiA-mediated i^6^A37 tRNA modification to improve translation and stability of RpoS (Aubee et al., 2016). Additionally, the *hfq* ORF is rich in leucine codons and loss of *miaA* also resulted in decreased expression of this protein, suggesting that MiaA aids in translation of *rpoS* both indirectly by regulating sRNA-binding and directly by improving translation efficiency (Aubee et al., 2017). TrmL and TusA are also involved with RpoS translational regulation (Aubee et al., 2017). TrmL is a methyltransferase that modifies tRNA by 2’-*O*-methylation of cytidine or uridine at position 34 (C/U34m), an important modification in leucine tRNAs (Benítez-Páez et al., 2010; Liu et al., 2013). In addition to MiaA, TrmL was also shown to be necessary for expression of RpoS through leucine decoding. TusA, which is a sulfur-carrying protein that mediates the 2-thiouridine (S^2^U34) wobble-position tRNA modification (Ikeuchi et al., 2006), was shown to be important for the efficient translation of *rpoS* (Aubee et al., 2017). Another study biochemically confirmed the role of TusA by showing that absence of TusA resulted in decreased translation efficiency of *rpoS*, through loss of thiolation on Lys, Glu and Gln tRNAs (Yildiz and Leimkühler, 2021). Thus, tRNA modifications and encoding of ORFs with specific codon preference, especially leucine, provide another means of regulation of RpoS, by modulating translational efficiency. It is unknown whether MiaA, TrmL and TusA are themselves regulated by stress factors, but their importance for effective RpoS, Hfq and RpoS target gene translation indicates that they could be yet another node for signal transduction in the GSR.

In addition to tRNA, there is evidence that rRNA usage might also play a role in regulation of RpoS. *E. coli* encodes seven rRNA operons (Blattner et al., 1997) which are highly similar and constitutively expressed (Condon et al., 1992), but which differ in sequence at certain conserved positions in each of the three ribosomal RNA genes. Thus, the pool of ribosomes in exponentially growing unstressed cells is heterogeneous, not identical. Kurylo et al. (2018) showed that *rrsH*, the 16S rRNA gene from the *rrnH* operon, is preferentially upregulated during nutrient limitation and increases RpoS protein levels. Furthermore, genes upregulated by *rrsH*-bearing ribosomes are also enriched for rare codons, similarly to the *rpoS* ORF. This suggests that the positive effect of *rrsH*-bearing ribosomes on RpoS may be due to improving the efficiency or fidelity of translation of *rpoS*, potentially through the sequence variants in the small ribosomal subunit head domain (Kurylo et al., 2018; Majdalani et al., 2023). Furthermore, RpoS levels have also been monitored at the single-cell level. This study showed that the RpoS-levels between the single cells of *E. coli* are extremely heterogenous, and that this heterogeneity arose from RpoS-pulsing and mutual inhibition between RpoS and growth rate. Thus, this adds another layer of regulation of RpoS, since this heterogeneity of activity of RpoS could function as a kind of stress anticipation between single cells, leading to subpopulations of cells that are better suited for responding to stress (Patange et al., 2018). Through ChIP-seq and RNA-seq, it has also been demonstrated that genes in the RpoS-regulon vary in their sensitivity to RpoS levels. Functionally related genes often had similar patterns of sensitivity, suggesting that sensitivity can act as a control mechanism to coordinate responses to specific stressors. In other words, RpoS sensitivity correlates with the function of the gene in the GSR. RpoS-sensitive genes included *arcA* and *rssB*, suggesting that response to energy-status and feedback regulation of RpoS-protein levels are highly prioritized compared to other stressors (Wong et al., 2017).

In addition to these alternative means of regulation of RpoS, sigma factor competition for the core RNA polymerase enzyme also plays a major regulatory role. This form of regulation was not touched upon here, but interested readers are referred to a recent review of this topic by Nandy (2022).

## Conclusion

**Figure 2:**
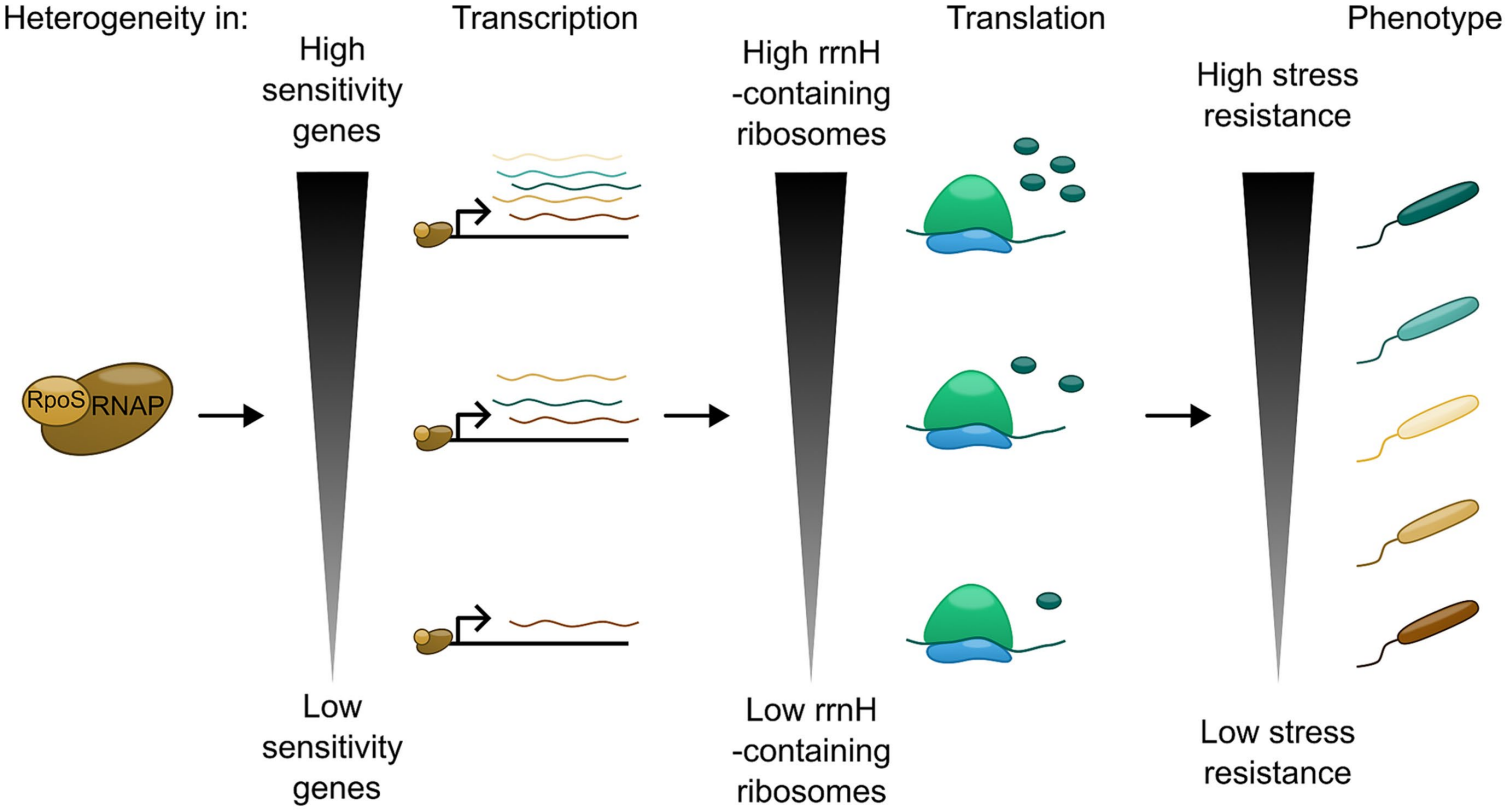
RpoS association with RNAP can lead to a heterogeneous phenotypic response through transcriptional and translational mechanisms.

Although the induction of RpoS leads to the same response of upregulating the genes for the GSR for *E. coli* to survive stationary phase, the outcome of this response may vary. Not only is this sigma factor sophistically regulated at multiple levels to balance all possible signal inputs, but also capable of fine-tuning the gene expression output to match the needs of the bacteria in the specific environment. Within a single cell, RpoS target genes have different sensitivities to RpoS concentration, which would lead to differing composition of the stress-induced proteins depending on the source of the stress. At population level, heterogeneous RpoS levels between cells, arising from different rRNA compositions of bacterial subpopulations, could result in generation of cells that are more suited to respond to the given stress than others (Patange et al., 2018). Hence, RpoS-mediated GSR activation is not a binary gene-expression switch, but is able to mediate phenotypic heterogeneity in the stressed population (Figure 2). This effect would likely increase evolutionary fitness for a subset of the population, promoting its long-term survival. Further exploration of GSR-induced heterogeneity will likely be highly relevant for understanding of pathogenesis and disease treatment in *E. coli* and related bacteria.

## Author contributions

SH: Visualization, Writing – original draft, Writing – review & editing. CLK: Funding acquisition, Visualization, Writing – original draft, Writing – review & editing.
